# Revised Ethics Guidelines for Environmental Epidemiologists

**DOI:** 10.1289/ehp.1205562

**Published:** 2012-08-01

**Authors:** Shira Kramer, Colin L. Soskolne, B. Adetoun Mustapha, Wael K. Al-Delaimy

**Affiliations:** 1 Epidemiology International Inc., Hunt Valley, Maryland, E-mail: s.kramer@epiinternational.com; 2 School of Public Health, University of Alberta, Edmonton, Alberta, Canada; 3 Department of Epidemiology and Biostatistics, MRC-HPA Centre for Health and Environment, Imperial College, London, United Kingdom; 4 Division of Global Health, Department of Family and Preventive Medicine, University of California, San Diego, San Diego, California

Environmental epidemiology, a well-established subspecialty of epidemiology, is of vital importance in the identification and prevention of environmentally linked morbidity and premature mortality. Perhaps more than most other applied sciences, the discipline of environmental epidemiology faces significant ethical challenges because of the involvement of powerful stakeholders whose influence may affect all levels of research and policy formulation. Although findings of environmental epidemiologic studies play a critical role in arguing for evidenced-based policies aimed at protecting the public from harms, they can also have direct effects on industry profits, political careers, academic funding, and professional advancement. Conflicting interests among various stakeholders create ethically challenging situations that may threaten the core tenets of the discipline, which focus on maintaining, enhancing, and promoting health in communities worldwide.

The International Society for Environmental Epidemiology (ISEE) Ethics and Philosophy (E & P) Committee is one of the earliest, active, and enduring ethics committees in the field of epidemiology. Since its inception in 1991, the committee has taken an active role in supporting ethical conduct and formulating ethics guidelines for the profession of environmental epidemiology, publishing its first set of Ethics Guidelines in 1996 (Soskolne and Light 1996) and formally adopting the guidelines in 1999. These guidelines addressed the four major categories of ethical conduct: obligations to subjects of research, obligations to society, obligations regarding funders/sponsors and employers, and obligations to colleagues.

Our profession functions within a social and political context in which laws, technology, economic pressures, and social norms are evolving. Thus, it behooves the custodians of guidelines to revisit their guidelines from time to time to reflect on new ethical challenges and on the current context in which they are to be applied. We recognize that guidelines cannot be enforced; they serve rather as a reference and pathway for ethically conscious professionals in our field who are seeking to improve the integrity of their research or to resolve ethical challenges that they face in their work.

At the 2009 ISEE annual meeting in Dublin, Ireland—10 years after the Ethics Guidelines were adopted—a subcommittee of the ISEE E & P committee was formed to review and update the guidelines. Certain trends and growing research challenges served as an impetus for the project, including

A sharp increase in reports of conflicting interests (> 7,000 references in a 2011 PubMed search)
An increase in industry-funded research at academic institutions (and a concomitant increase in the proportion of academic faculty supported by or funded by industry)
Research with an *a priori* agenda and expected results
Industry and economic stakeholder influence on government policies
Environmental (in)justice
Imbalances in allocation of research funding and priorities
Purposes of environmental epidemiologic research
Public input into public health research processes and policies
Data access and ownership of public health information
Nature of informed consent
Control and use of biospecimens
Maintenance of confidentiality
Fair attribution for research contributions and protection of intellectual property
Influence of ghost-written scientific articles on law making and on international bodies whose mission is to protect the public interest
Challenging unacceptable behavior and protecting whistleblowers
Major expansion of the field of environmental epidemiology
The globalization of public health issues that require collaborative professional efforts to address them.

The goals of the subcommittee focused on including current and evolving ethical and philosophical challenges, re-crafting recommendations and providing guidance for professional conduct, and providing case studies to enhance the relevance of the guidelines and to stimulate discussion about solutions (still pending). A core writing group (S.K., C.L.S., B.A.M., and W.K.A.D.) took primary responsibility for conducting the review and developing revisions, with input invited from members of the E & P committee at large. Over a 2-year period, and through an iterative process, suggested revisions and updates were evaluated and incorporated by consensus of the core writing group and the E & P committee.

Once the draft guidelines were accepted by majority vote of the E & P committee, they were submitted to the Governing Council of the ISEE for comment and review. Members of the Governing Council submitted additional comments and suggested changes, leading to further refinement by the core writing group. The finalized version of the *Ethics Guidelines for Environmental Epidemiologists* was accepted and adopted by the Governing Council of the ISEE on 25 April 2012 [ISEE 2012; see also Supplemental Material (http://dx.doi.org/10.1289/ehp.1205562)]. This is the first time that the guidelines (first published in 1996) have been revised.

## Key Components of the Guidelines

The aim of this editorial is to introduce readers to the guidelines by highlighting key ethical topics for environmental epidemiologists. By describing the concepts covered in the guidelines, we provide a ready reference for our colleagues as well as for the general public.

The Ethics Guidelines are structured into four major sections with key subsections. The structure of the four topics has not changed from the original guidelines because these four areas of ethical consideration remain foundational to environmental epidemiology research and practice. The four obligations are to individuals, to society, to funders and employers, and to colleagues.

### Obligations to Individuals and Communities Subjected to Research

Epidemiologists and supporting institutions are obliged to recognize the rights of research participants. This expectation is not unique to environmental epidemiology; it reflects standard bioethical principles.

Four primary themes delineate these obligations:

***Research should avoid harm to the individuals and communities studied. Knowledge gained should be disseminated widely, and benefits gleaned should be accessible to the community studied.*** This topic covers the concepts of *a*) beneficence (i.e., doing good), *b*) the precautionary principle, *c*) nonmaleficence (i.e., doing no harm), *d*) respect for autonomy (i.e., the individual’s right to self-determination), *e*) community input in the research process, *f* ) full disclosure of risks and benefits, and *g*) prompt disclosure of results.
***Informed consent before research is initiated.*** This core ethical consideration addresses *a*) individual rights, *b*) public communication, *c*) consent for biospecimens, *d*) cultural sensitivity of consent, *e*) financial disclosure of all sources of financial support, *f* ) financial conflict verification, and *g*) confidentiality of public data and records.
***Confidentiality.*** A framework for assuring confidentiality includes the need for a confidentiality plan and data security, avoiding identification of individual participants, sharing of confidential information, and extraordinary circumstances where breach of confidentiality may be justified.
***Review of research protocols by institutional review boards (IRBs) or equivalent oversight committees.*** The critical role of IRBs, or their equivalent, in the review and oversight of research is discussed, including *a*) IRB roles and responsibilities, *b*) local values in ethics oversight, *c*) ethics and study design, *d*) principal investigator’s responsibility for ethical practice, and *e*) conflicting interests of IRB reviewers.

### Obligations to Society

As public health professionals, we emphasize epidemiologists’ obligations to society. The guidelines address several important ethical considerations that may affect this fundamental responsibility:

***Avoiding partiality.*** Whether conscious or unconscious, partiality should be avoided, impacting the choice of research methods and communication of results, inappropriate interference in research, and avoidance of bias.
***Avoiding conflicting interests.*** There is a growing threat to research integrity fueled by conflicting interests. This section emphasizes the need to avoid conflicting interests, provide full disclosure of financial or other relationships, and ensure transparency in disclosures.
***Conduct that facilitates just environmental health policy and practice.*** We acknowledge the need for *a*) recognition of different ethical worldviews, the *b*) precautionary principle and its role in causal inference, *c*) contextualization of research results, *d*) guidelines for reanalysis of data, *e*) advocacy, *f* ) distributive justice, *g*) research priorities as a reflection of public health burden, and *h*) issues surrounding data access.
***Community involvement.*** The key aspects of community involvement focus on engagement of stakeholders, partnerships, and conveying information of uncertain biological significance.
***Communication and action plan.*** This aspect of research practice includes *a*) reporting of research findings, *b*) communication with the media, *c*) transparency regarding assumptions and uncertainties, *d*) communications and action plan, *e*) avoidance of misrepresentations and improper inferences, and *f* ) psychological impact of research results.

### Obligations Regarding Funders/Sponsors and Employers

There is sometimes a tension between the interests of various stakeholders and the primary public health goals of environmental epidemiologic research. The guidelines address core principles that may serve as a guide in these circumstances.

**Specifying obligations.** In order to protect research integrity, we should evaluate the motivations of stakeholders in order to protect the public interest, communicate obligations to funders and employers, and avoid funding or other undue influence on research methods or results.
**Protecting privileged information (including intellectual property and trade secrets).** Privileged information may be used in the conduct of research, provided that permission is granted and confidentiality restrictions are maintained.

### Obligations to Colleagues

As members of a diverse research community, we should maintain respect and fairness toward colleagues. Often, these issues are the most difficult to confront because they may affect personal and professional relationships. The guidelines highlight key considerations, including

**Specifying obligations.** The guidelines address the importance of respect for intellectual property and research ideas, fair attribution, avoidance of conflicting interests, and misappropriation of research ideas.
**Reporting methods and results.** Reporting should enable assessment and replication of results, allow independence and neutrality, be subjected to peer review, and support objectivity of reviewers.
**Confronting unacceptable behavior.** Appropriate means of confronting improper practices among colleagues are supported, including the role of international review panels to review alleged misconduct, and protection of whistleblowers.
**Communicating ethical requirements among colleagues and other stakeholders.** Ethical requirements that are applicable to research and practice should be shared with colleagues, research staff, funders, and practitioners.

## Incorporating ISEE Ethics Guidelines into Training and Practice

These guidelines are meant to provide a framework, rather than a set of rules or an ultimate solution, as we confront ethical tensions. Indeed, ethics are relative, and some concepts that are acceptable in Western societies, for example, might not be relevant or applicable in other cultures. Guidelines provide practical approaches that can help maintain the fundamental tenets of our discipline and provide thoughtful researchers and practitioners a point of reference for decision making in an environment laden with complex pressures.

The benefits of the ISEE Ethics Guidelines will be realized only if they are relevant to members of our profession and widely disseminated. We believe that relevance would be enhanced if there were an ongoing mechanism for submitting and appending real-life case studies that illustrate actual occurrences of tensions or conflicts and their resolution. The next phase of this project will be to provide a framework for members of the ISEE to submit case studies in a standard format and make them widely available. The case studies will be valuable for teaching, as well as a means of communication and support within the profession about sensitive, isolating, and even threatening circumstances that can occur within our research community. Members of the ISEE E & P Committee are currently working on this phase of the project.

New generations of researchers and professionals are becoming more aware of and interested in this discipline and its impact on their work; they recognize that research ethics can no longer remain on the fringes of scientific discourse. Ultimately, the value of this work will be realized only if the ISEE Ethics Guidelines are actively incorporated into training programs, included in institutional practices and standards, integrated into presentations or discussions at professional meetings, and promulgated as a constructive set of principles to protect the integrity and values of the profession (Soskolne and Sieswerda 2003).
